# Internet Use among Ugandan Adolescents: Implications for HIV Intervention

**DOI:** 10.1371/journal.pmed.0030433

**Published:** 2006-11-07

**Authors:** Michele L Ybarra, Julius Kiwanuka, Nneka Emenyonu, David R Bangsberg

**Affiliations:** 1 Internet Solutions for Kids, Irvine, California, United States of America; 2 Mbarara University of Science and Technology, Department of Pediatrics, Mbarara, Uganda; 3 Epidemiology and Prevention Interventions Center, Division of Infectious Diseases, San Francisco General Hospital, University of California San Francisco, San Francisco, California, United States of America; 4 The Positive Health Program, San Francisco General Hospital, University of California San Francisco, San Francisco, California, United States of America; University of Connecticut, United States of America

## Abstract

**Background:**

The Internet is fast gaining recognition as a powerful, low-cost method to deliver health intervention and prevention programs to large numbers of young people across diverse geographic regions. The feasibility and accessibility of Internet-based health interventions in resource-limited settings, where cost-effective interventions are most needed, is unknown. To determine the utility of developing technology-based interventions in resource-limited settings, availability and patterns of usage of the Internet first need to be assessed.

**Methods and Findings:**

The Uganda Media and You Survey was a cross-sectional survey of Internet use among adolescents (ages 12–18 years) in Mbarara, Uganda, a municipality mainly serving a rural population in sub-Saharan Africa. Participants were randomly selected among eligible students attending one of five participating secondary day and boarding schools in Mbarara, Uganda. Of a total of 538 students selected, 93% (500) participated.

Of the total respondents, 45% (223) reported ever having used the Internet, 78% (175) of whom reported going online in the previous week. As maternal education increased, so too did the odds of adolescent Internet use. Almost two in five respondents (38% [189]) reported already having used a computer or the Internet to search for health information. Over one-third (35% [173]) had used the computer or Internet to find information about HIV/AIDS, and 20% (102) had looked for sexual health information. Among Internet users, searching for HIV/AIDS information on a computer or online was significantly related to using the Internet weekly, emailing, visiting chat rooms, and playing online games. In contrast, going online at school was inversely related to looking for HIV/AIDS information via technology. If Internet access were free, 66% (330) reported that they would search for information about HIV/AIDS prevention online.

**Conclusions:**

Both the desire to use, and the actual use of, the Internet to seek sexual health and HIV/AIDS information is high among secondary school students in Mbarara. The Internet may be a promising strategy to deliver low-cost HIV/AIDS risk reduction interventions in resource-limited settings with expanding Internet access.

## Abstract 2

A survey among 500 adolescent pupils in rural Uganda suggests widespread interest in online information about sexual health and HIV/AIDS. Over one-third of Internet users had already searched for relevant information online, and many of the others said they would like to educate themselves about HIV/AIDS online.

## Editors' Summary

**Background.:**

HIV/AIDS is a major health burden in sub-Saharan Africa, including Uganda. Despite a recent reduction of the number of HIV-infected individuals, HIV transmission remains a problem among Ugandan adolescents. Recent surveys suggest that about half of sexually active adolescents do not consistently use condoms, and that young people are less knowledgeable about HIV than they were 15 years ago.

**Why Was This Study Done?:**

The Internet has a number of characteristics that make it an attractive tool in health education and HIV prevention, especially for adolescents—including interactivity, privacy, the overlap between education and play, and the ability to individualize information based on an initial assessment of background conditions, interest, and knowledge. It is also thought that despite these advantages, the Internet's potential in resource-poor settings with higher HIV infection rates and limited access to other health care resources has not been explored much. This study was done to gain some initial insights on the desired and actual use of the Internet to seek sexual health and HIV/AIDS information among adolescents in Uganda.

**What Did the Researchers Do and Find?:**

They did a survey of 500 adolescent pupils randomly selected from five participating boarding schools in Mbarara, a small town in a rural part of Uganda. They asked three questions: To what extent are the adolescents exposed to computers and the Internet? Are they interested in accessing health information online? Who uses the Internet and how? Almost half of the participants said they had used the Internet at least once, and the majority said they had been online during the previous week. Most Internet users (82%) reported going online at school; 57% said they use Internet cafes, 17% access the Internet at home; and 11% at someone else's house. More than a third of all participants reported having used the Internet or computer to look up health information, and many had been looking for information on sexual health and HIV/AIDS. About two-thirds of the participants said that if Internet use were free, they would search for information on sexual health and HIV/AIDS prevention. The researchers analyzed the responses further to identify the most influential factors in whether one of the Internet users would go online to educate themselves about HIV/AIDS. They found that those participants who used the Internet more often and those who engaged in online activities like chat rooms, games, and e-mail, were more likely to search for HIV/AIDS information. On the other hand, those who went online only at school were less likely to do so.

**What Do These Findings Mean?:**

Approximately the same proportion—roughly one-third—of adolescents in a rural setting in Uganda reported having used the Internet to look up health-related information as of young people in the United States. Together with the result that an additional third said that they would go online to educate themselves about HIV/AIDS if Internet use was free, this study suggests that initiatives in Africa to improve online access for adolescents as well as to develop content tailored for young people in specific settings would make a difference.

**Additional Information.:**

Please access these Web sites via the online version of this summary at http://dx.doi.org/10.1371/journal.pmed.0030433

- Links page for adolescents and youth from HIV InSite at UCSF
- Africa Initiative
- HIV/AIDS education module from the US Public Broadcasting System
- Lesson plan for “Using the Internet to Access Sexual Health Information”  from the Information Institute of Syracuse

## Introduction

HIV/AIDS is a major contributor to morbidity and mortality in sub-Saharan Africa, including Uganda [1]. While a government-led public health campaign has dramatically decreased HIV prevalence from an estimated 15% in 1991 to 5% in 2001 [2], recent statistics suggest that HIV transmission remains a problem among adolescents. Estimates based on studies in pregnant women reveal that 10% of people between the ages of 15 and 24 years were HIV-positive in Kampala in 2001 [3]. Of sexually active men and women between the ages of 15–24 years, respectively 38% and 56% did not report using a condom at last higher-risk sex [1], and HIV knowledge appears to be on the decline compared to young people surveyed in 1990 [3].

The Internet is recognized as a valuable tool for intervention and prevention in Western cultures [4,5]. This may be especially true for adolescents, who have overwhelmingly adopted the technology as another environment in which they interact and learn [6]. An estimated 25%–31% of young people in the United States have looked for health information online in the last year [7,8]. Common topics are those that are “hard to talk about” including sexual health, depression, and drug use [7,9]. Currently, Internet-based interventions are addressing hard-to-treat conditions such as smoking [10–13] and major depressive disorder [14,15], with adolescent-focused HIV prevention Web sites also in development [16].

Access to reliable disease information online is not only informative, but has been linked to positive change. Among young people, two in five (41%) have changed their behavior because of information they found online [17], and almost half (49%) have contacted a health care provider as a result [8]. The Internet also overcomes many of the potential challenges of traditional interventions, including competition for time and resources (e.g., in schools, sex education is competing for classroom time with other curricula), facilitator issues (i.e., variance in fidelity of implementation or concern/embarrassment with some topics), and rigidity of content and design [4]. Unlike traditional interventions, Internet-based interventions are widely accessible across broad geographic areas, are inexpensive to scale up, and allow users to identify a stigma-free, anonymous atmosphere in which to receive individually tailored information [4].

Recent data suggest explosive Internet growth in resource-limited settings. The number of Internet users in Uganda increased from 1.6 per 1,000 in 2000 to 7.2 per 1,000 in 2004 [18]. A total of 2,496 Internet hosts were providing Internet access to 200,000 users in 2005 in Uganda [19]. Anecdotal reports suggest that Internet access is available in almost all of Uganda's major urban centers [20,21].

Despite the advantages of the Internet as an intervention delivery method, its feasibility is unknown in resource-limited settings with higher HIV prevalence and more limited health care resources [22]. To better understand the potential to deliver adolescent, Internet-based health interventions in a resource-limited setting, we addressed the following three questions. (1) What is the current exposure to computers and the Internet among secondary school students in a small urban trading center? (2) What is the extent of interest in accessing health information online? (3) What are the demographic characteristics of Internet users and how do they use this technology?

## Methods

### Location and Participants

Mbarara municipality, with a population of 69,000 (based on the 2002 census), is the sixth largest urban center in Uganda [23]. The greater Mbarara district is second in population only to the Kampala district, yet it falls in the bottom half of districts in terms of population density. Mbarara municipality is therefore best described as serving mainly a rural population in sub-Saharan Africa. Access to education in Mbarara is mixed. Mbarara University of Science and Technology is highly regarded and is the 18th largest of Uganda's 53 tertiary institutions [23]. Data at the district level indicate that Mbarara district's 2004 net secondary enrollment rate was slightly lower than the national average (11.3% versus 14.6%) [23]. HIV prevalence rates in Mbarara were estimated to be 10.8% in 2002, which is well above the 2003 country average of 4.1% and the second-highest rate reported outside of Kampala [1].

Participants in the Uganda Media and You survey were recruited from five day and boarding schools in Mbarara municipality; the students who attend these schools represent diverse demographic characteristics (Table 1). Three schools were government-sponsored (public), one was run by a Catholic religious organization, and another was run by a Muslim religious organization. All five schools provide education to students in classes Secondary 1 though Secondary 6 (roughly equivalent to grades 8–13 in the United States).

**Table 1:**
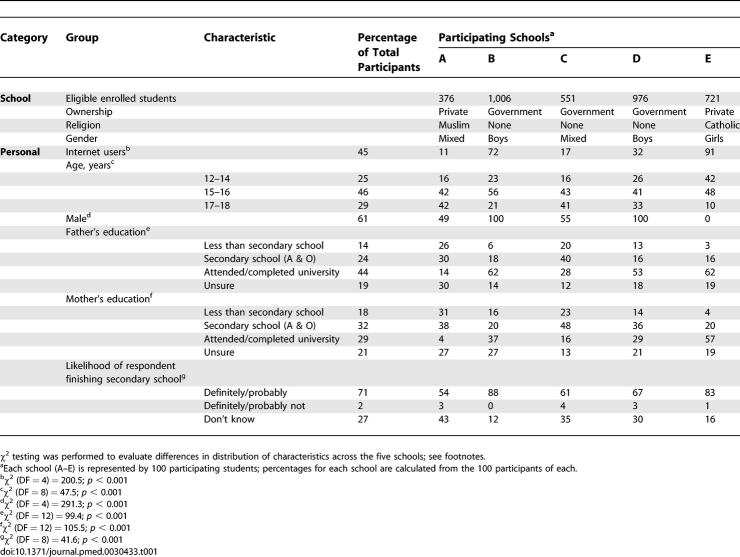
Survey Sample Characteristics

Headmasters provided the project director (NE) with an alphabetical list of all eligible participants. All students (*n* = 3,630) enrolled in grades Secondary 1 through Secondary 4 (roughly equivalent to grades 8–11 in the United States) at one of the five participating schools were eligible. From each school 100 participants were randomly selected using a computer-generated list of random numbers (http://www.randomizer.org/form.htm). At three of the five schools, 100% (300) of invited participants chose to participate. At the remaining two schools, 38 students (13 from one, and 25 from another) were either absent or declined to participate. These students were replaced by those listed next on the random list; all 38 additional students contacted next agreed to participate in the survey. Of 538 eligible and identified to participate, 93% (500) completed the survey.

At the designated time after school, students randomly identified to take the survey went to the school's general assembly hall. Surveys were completed in the absence of the teachers and school administrators. On average, the survey took 45 minutes to complete.

Informed consent was obtained from the School Headmasters, and all student participants provided written informed assent. IRB approval for the study was received from the Mbarara University Institutional Ethical Review Committee.

### Measures

Participants first were asked what resources they used for various research activities, including school work, general health information, sexual health information, and HIV/AIDS information. Next, they were asked about Internet use, including whether they or someone they knew had ever used the Internet. For those who acknowledged personal Internet use, questions were asked about where they accessed the Internet, activities they engaged in while online, and the frequency and intensity of Internet use. Those who indicated that they had never used the Internet were asked to indicate their reasons (e.g., did not want to, parents would not allow it, etc.). Finally, all respondents were asked to respond to several Internet use questions given a hypothetical scenario that Internet access was free, including posited frequency of use as well as whether they would search for specific health topics. In all cases, in addition to multiple choices, respondents were offered an “other” option where they could write in their response.

### Statistical Methods

Missing data and “don't know” responses for demographic characteristics were coded to the sample mode unless deemed informative, in which case they were combined into a specific category. All other data (e.g., exposure to the Internet) were conservatively coded as symptom absent. After reporting descriptive statistics, logistic regression was used to estimate the odds of reporting Internet use, adjusting for underlying differences in demographic and school variables (adjusted odds ratio [AOR]). Similarly, logistic regression was used to compare usage characteristics among Internet users by sex and age, adjusting for school characteristics and all other demographic characteristics. In some cases, school attended was highly collinear with the explanatory variable of interest. When this happened, the three defining school characteristics (i.e., size, male-female ratio, private versus public management) were investigated separately, and the collinear subvariable was dropped. Finally, to identify the most influential characteristics related to the odds of using the computer or Internet to look for HIV/AIDS information, a parsimonious logistic regression model was identified via backward stepwise regression techniques and confirmed using forward stepwise regression [24]. A saturated model was estimated, including demographic characteristics, school attended, Internet log-in location, frequency of Internet use, and activities online. Variables that were not significant (*p* > 0.05) were dropped singly from the model, resulting in a parsimonious model of the most influential characteristics in creating a profile of HIV/AIDS Internet information seekers.

## Results

### Study Sample

As shown in Table 1, the average survey respondent was male (61% [304]) and between 15 and 16 years of age (46% [230]). All respondents were African. Forty-four percent (219) of participant's fathers were reported to have attended university, as were 29% (143) of participant's mothers. Forty-one percent (*n* = 206) of respondents indicated that they would definitely finish secondary school, while an additional 30% (147) indicated that it was likely. Because so few students indicated that their likelihood of finishing school was “definitely/probably not,” this category combined with “don't know” for subsequent analyses.

Internet use varied significantly by school (χ^2^ [DF (degrees of freedom) = 4] = 200.5; *p* < 0.001); exposure ranged from 11% to 91% across the five source schools. The two schools with the highest percentage of Internet use were same-sex: One was an all-boys, publicly administered school, and the other was an all-girls private school.

### Current Exposure to the Internet among Adolescents Attending Secondary School in Mbarara

The Internet was reported as used at least once in a lifetime by 45% (223) of respondents. An additional 43% (214) of adolescents knew someone, such as a family member or friend, who had used the Internet. Internet use was frequent: Of the 223 Internet users, 78% (175) reported going online in the previous week and 41% (91) were online for 1 hour or longer the last time they had logged on. The majority of these Internet users (82% [182]) reported going online at schools, with a large percentage also going online at Internet cafes (57% [127]). Almost one in five reported going online at home (17% [39]), while one in ten reported going online at another house (11% [24]).

### Extent of Interest in Accessing Health Information Online

More than one in three of all respondents (38% [189]) reported already having used computers or the Internet to look up health information. Additionally, 35% (173) reported using the computer or Internet to find information about HIV/AIDS, and 20% (102) had looked for sexual health information. The majority of all respondents reported that, if Internet access were free, they would search for information about sexual health (61% [307]) and HIV/AIDS prevention information (66% [330]). Reasons cited for using the Internet as a health information resource if it were available for free included the vast amount of information available (72% [358]) and privacy (28% [138]).

### Demographic Characteristics of Internet Users

As shown in Table 2, participants who reported that their mothers had attended secondary school were 2.4 times as likely (*p* = 0.03) and those whose mothers had attended university were 4.4 times as likely (*p* = 0.002) to report Internet use when compared to participants whose mothers had completed less than a secondary school education (after adjusting for differences in school attended and demographic characteristics [i.e., likelihood of finishing secondary school, paternal education, age, and sex]). An opposing trend was suggested for father's education, although adjusted estimates were not statistically significant.

**Table 2:**
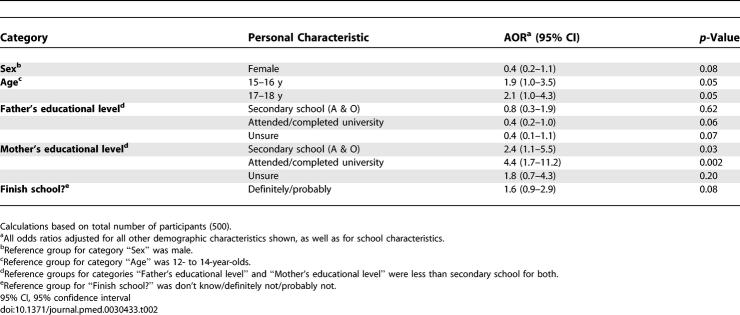
Logistic Regression Model Estimating the Odds of Internet Use Based upon Demographic Characteristics

Participants who were 15–16 years old were twice as likely (*p* = 0.05) as were those 17–18 years old (*p* = 0.05) to report Internet use versus otherwise similar 12- to 14-year-old respondents.

A nonsignificant trend was suggested for females as less likely to use the Internet than males after adjusting for all other demographic and school characteristics.

As shown in Table 3, almost all Internet usage characteristics were similar among male and female Internet users. There was however, a strong suggestion that female Internet users may be more likely than otherwise similar male Internet users to go online at school and at home. Boys who used the Internet were significantly more likely to play games online, whereas a nonsignificant trend suggested girls were more likely to use email.

**Table 3:**
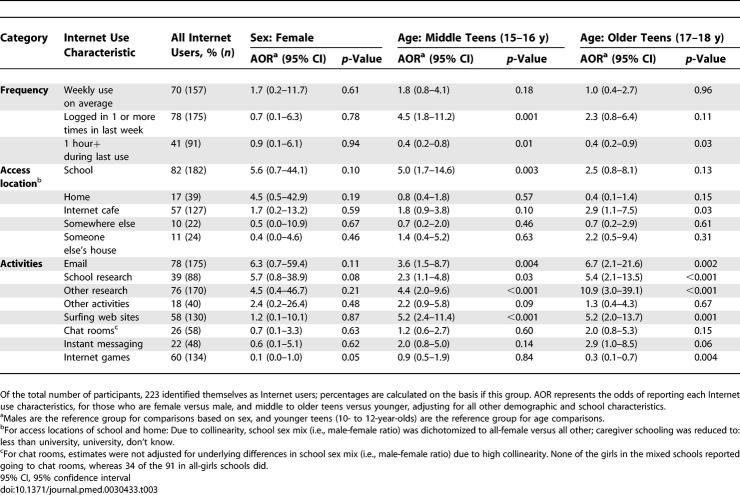
Estimated Odds of Internet Use Characteristics Based on Participant Sex and Age

Important differences in Internet usage were observed across age groups. When compared to otherwise similar younger teenagers (12- to 14-year-olds), middle teens (15- to 16-year-olds) were 4.5 times as likely to report using the Internet one or more times in the previous week versus not (*p* = 0.001), with similarly elevated but nonsignificant trends for older teens (17- to 18-year-olds). Yet, middle teens and older teens were half as likely to report going online for 1 hour or longer versus a shorter period when compared to younger teens.

To help direct the development of future HIV/AIDS prevention efforts, a parsimonious logistic regression model was estimated to identify the most influential characteristics in predicting the odds of using the computer or Internet to look for HIV/AIDS information among current Internet users (Table 4). First, collinearity was estimated within the saturated model. Indications of high collinearity between school attended and Internet use were noted. Subsequent examination revealed collinearity between being male and the sex composition of source schools among Internet users. As such, the variable reflecting school sex mix was dropped from the saturated model. Both remaining school characteristics (school size and ownership type) were forced into the final model irrespective of statistical significance. Acceptable model fit (Hosmer-Lemeshow χ^2^ [DF = 6] = 2.4; *p* = 87.6) was observed for the parsimonious model. The global variable reflecting school attended was added to the final model to observe any potential influence it had on estimates; little influence was observed.

**Table 4:**
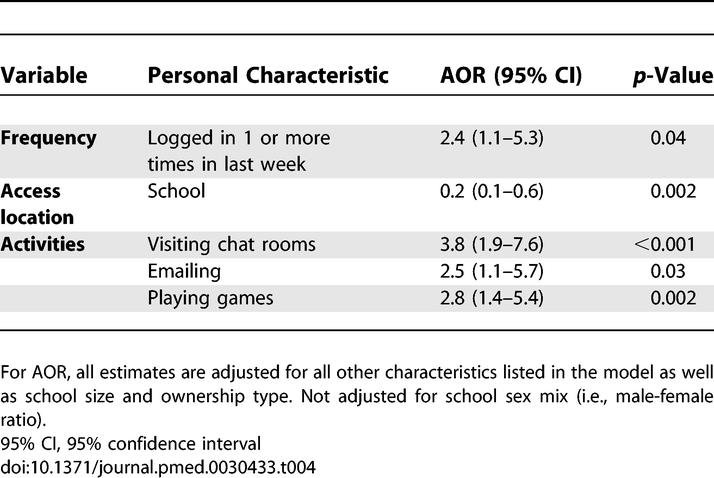
Parsimonious Logistic Regression Model for HIV/AIDS Information Seekers among Internet Users (*n* = 223)

As shown in Table 4, young people who used the Internet in the previous week were 2.4 times more likely as to be HIV/AIDS information seekers as compared to otherwise similar Internet users who had not logged on within the previous seven days (*p* = 0.04). Online activities, specifically visiting chat rooms, utilizing email, and playing games, were each strongly associated with HIV/AIDS information-seeking among Internet users after adjusting for all other influential characteristics. Conversely, using the Internet at school was associated with 80% lower odds of HIV/AIDS information seeking among otherwise similar Internet users (*p* = 0.002). No other characteristics, including sex, age, and parental education, were influential in predicting HIV/AIDS information-seeking behavior.

### Reasons for Not Using the Internet

Among the 55% (277) of participating adolescents who had never used the Internet, the most common reason cited was its expense (43% [118]). Over one-third of respondents also indicated not knowing where to access the Internet (39% [107]) and not knowing how to use the Internet (36% [101]). Less than 15% of non-Internet users reported they did not use the Internet because of the slow connection speed (12% [33]), parental rules against Internet use (8% [23]), or lack of desire to go online (9% [25]). Free responses (11% [30]) also included time issues (e.g., “it needs some time yet I would like to learn” [7]) and general access constraints (e.g. “no access” [8]).

## Discussion

Public health researchers are beginning to embrace the Internet as a new, interactive technology with potential for use as an intervention tool in developed countries [4], yet little research attention has been paid to similar applications in Africa. Indeed, while the Internet is as an important resource to connect academicians and physicians in Africa to current medical literature [25–28], its value as a patient tool is yet unknown. Our data suggest that nearly half (45% [223 of 500 participants]) of adolescents attending secondary school in Mbarara, a municipality serving mainly a rural population in Uganda, use the Internet. An additional 43% (214) know someone who had gone online. Interest in using technology as a healthcare information resource appears high: 66% (330) of all secondary school respondents indicated that they would search for HIV/AIDS online if access were free. Adding validity to these estimates, a sizable minority (38% [189]) of all respondents already has used a computer or the Internet to look for information about sexual health, and 35% (173) had used these technologies to look for HIV/AIDS information specifically. These data suggest that respondents are familiar with the Internet, and more than one in three adolescents already are comfortable using the computer as a resource for health information.

### Participant and Venue Characteristics Related to Internet Use

Higher levels of maternal education are associated with increasing likelihood of Internet use among young people, whereas paternal education is not. This may suggest that mothers have a stronger influence on their children's education and environmental exposures than do fathers. It also may be an indication of a more liberal family environment in general, one of the manifestations of which is a more permissive or direct orientation toward new technologies such as the Internet. Further research is warranted to inform family-based intervention programs.

Females are more vulnerable to HIV infection because of a variety of biological, economical, and cultural reasons [29–31], and this is especially true in resource-poor countries where women generally are less empowered to make decisions about their sexual health [32–34]. Our data suggest that after adjusting for demographic characteristics and school attended, adolescent women may be somewhat less likely to report Internet use than are adolescent men (Table 2). Among Internet users, however, adolescent women seem equally likely to access the Internet frequently and are equally likely to be HIV/AIDS information seekers (Table 3). Furthermore, 91% of respondents from the participating all-girls secondary school have been online. Thus, it appears that any sex differences in Internet exposure may be less associated with interest or ability and may be perceived more accurately as an issue of access. Targeted efforts to ensure young women access to the Internet may be needed.

Among Internet users, accessing the Internet at school appears related to a significantly decreased likelihood of searching for HIV/AIDS information on a computer or the Internet after adjusting for all other significant characteristics (Table 4). This may be because young people are concerned about their privacy and are being monitored or in some other way feeling inhibited by school faculty. It also is possible that blocking software has been installed on computers, disallowing searches related to sexual behavior. Therefore, schools currently may be a promising place for recruitment for Internet-based interventions that can be reinforced by providing access to Internet cafes or home environments. Nonetheless, the Internet holds promise as a substitution for or adjunct to school-based adolescent HIV prevention education. Kinsman et al. [35] report that intervention-based activities most likely to reduce HIV risk behaviors—those about condoms and role playing—were covered by Ugandan teachers in their study only superficially because of not enough classroom time, their fear of potential controversy, and teachers feeling unfamiliar with the topic. School-based interventions remain compelling, however, because school is the easiest environment from which to reach the greatest number of young people. Structural and cultural changes that allow for greater privacy and less monitoring of Internet use for the specific purpose of successful implementation of school-based Internet prevention programs should be considered.

### Using the Internet to Tailor the Program to the Individual

Internet-based interventions are beneficial in their ease of tailoring to individual risk profiles. This is especially important during adolescence, which is a time of rapid biological development and changes in sexual experience. Current findings suggest that among Internet users, adolescent boys and girls across the age spectrum are equally likely to report looking for HIV/AIDS information on a computer or online. Prevention programs therefore can be developed for all adolescents and tailored to identified risk behaviors, allowing current sexual behavior to drive the content. Thus, for example, a sexually active 12-year-old and an abstinent 18-year-old can receive different tailored prevention information. To do so, a brief risk assessment could be offered at the beginning of the online intervention to gauge the adolescent's sexual behavior and HIV/AIDS knowledge. This assessment would drive the subsequent tailoring of prevention messages in order to highlight those HIV preventive issues most relevant to this adolescent's risk profile. Additional modules that educate about age- and sex-specific biological changes should be integrated, as could tailoring for reading level.

Among otherwise similar Internet users, females in the Uganda Media and You survey may be more likely to use email, whereas males are more likely to play games online (Table 3). Spending time doing either of these activities is strongly related to the odds of also using technology to look for information about HIV/AIDS (Table 4). This suggests the possible need to tailor the intervention design to sex-related activities. For content aimed at females, integration of online communication tools such as emailing may increase their involvement, whereas content aimed at males may be best delivered via interactive games. Similarly, 15–16 year old respondents were more likely than 12–14 year old respondents to report Internet access, with a similar trend noted for 17–18 year olds (Table 3). This Internet use characteristic also is strongly associated with using technology to look for HIV/AIDS information. Conversely, younger teens between the ages of 12 and 14 years are significantly more likely to report going online for 1 hour or more as compared to their otherwise similar, older peers. This suggests that the development of different frequency–intensity schedules may be an important aspect of tailoring. For example, users may choose between exposure to the same content in either six modules of 15 minutes each or 3 modules of 30 minutes each. Optimal timing (e.g., once a week versus every other day) should also be assessed during the developmental stages of the online intervention.

Bensely and colleagues [36] report that the Internet is an acceptable delivery method of HIV/AIDS health education among female adults in South Africa, yet there is a paucity of Web sites that are culturally appropriate and relevant for people living in resource-limited settings. In addition to tailoring to the characteristics of the user, the Internet-based prevention program must be culturally relevant and must acknowledge local issues affecting access to healthcare and preventive behaviors, such as differences in locally appropriate and available contraception. For example, access to condoms can be challenging for adolescents, especially in rural areas of Uganda. Some Ugandan health workers believe that providing condoms will encourage adolescents to have sex and refuse to distribute them to young people [37,38]. Supplies of free condoms often are unpredictable. Locally specific, up-to-date information about where to obtain condoms is necessary for sexually active young people to engage in effective HIV preventive behavior. Similarly, cultural issues such as financial and material transactions that can accompany sex must be acknowledged and targeted [35]. As with any new health promotion endeavor, focus groups and other formative, qualitative research efforts are needed to identify the HIV preventive information, motivation, and behavioral skills and deficits specific to the target population [39].

### Targeting Nonusers

Internet-based studies should include strategies to reach nonusers. Just over half of survey respondents (55% [277]) have not used the Internet, yet less than 10% of nonusers (5% of all respondents) report no desire to use the Internet. The most commonly reported barriers to Internet use among secondary school students in Mbarara who have not used the Internet are expense (43% [118]), not knowing where to access the Internet (39% [107]), and not knowing how to use the Internet (36% [101]). These data suggest that young people are interested in using the Internet but are unable to do so because of financial and knowledge barriers. Efforts aimed at providing free access and training would likely increase Internet use among those not currently online. Many current users report intensive Internet use and could be effective gatekeepers to exposing nonusers to the Internet.

### Limitations

Our findings should be appraised within the context of our study limitations. Mbarara municipality is the sixth largest urban center in Uganda and has one of the country's 53 tertiary educational centers. Current findings may not be generalizable to more rural settings with fewer educational resources, especially those that lack access to the Uganda national electricity grid. Nonetheless, our sample is one of the few that are based on respondents outside of a major capital in sub-Saharan Africa. Respondents likely reflect the first adopters of the Internet among adolescents in Uganda and as such, are important first clues to how technology can be used in future HIV/AIDS prevention/intervention efforts. Additionally, questions about HIV risk behavior and knowledge were not asked in the Media and You survey. This information might be integral in assessing the potential impact of Internet-based interventions, as well as their ability to reach those at greater risk for HIV. Future research should focus on this important question.

### Implications

Although data have not been systematically collected across districts in Uganda to allow comparisons of Internet access in Mbarara to other areas, anecdotal reports suggest that Internet access is available in almost all of Uganda's major urban centers [20,21]. Internet access likely is more prevalent in Mbarara than in smaller districts, but it is reportedly less prevalent than in larger districts such as Kampala and Jinja. Mbarara thus likely reflects communities on the cusp of Internet integration. If true, Mbarara is representative of the handful of municipalities that currently have Internet access in Uganda, and may represent a similar transition that will occur in smaller municipalities over the next few years. The differential in access to the Internet likely will decrease over time, as was seen in resource-rich settings [6], and similar to mobile phone use in resource-limited settings. Because Mbarara may be on the leading edge of Internet adoption, it serves as a useful case study of how Internet attitudes and behaviors change over time. Further research is warranted to track the ongoing infusion and growing influence that the Internet, and access to health information online, will have on young Ugandans across a broader geographic and socioeconomic spectrum.

Given the promising indications from this study that young people are interested in accessing HIV/AIDS preventive information over the Internet in the Uganda Media and You survey, public policy initiatives could be developed to accelerate broader Internet access. For example, providing free Internet access to all adolescents via state-owned or regulated providers could be a way of engaging young people in a larger public health HIV/AIDS preventive campaign. Just as the Gates Foundation and other technology leaders have promoted Internet access in schools in the United States, business leaders and governments could do so in developing countries. With expanding access to the Internet [18,19], online delivery of HIV/AIDS preventive interventions to adolescents living in resource-limited settings appear promising.

## Supporting Information

Alternative Language Abstract S1 Spanish Translation of the Abstract by Michele Ybarra (23 KB DOC)

## Abbreviations

AOR: adjusted odds ratio
DF: degrees of freedom
