# Supplementary material for: Internet Use among Ugandan Adolescents: Implications for HIV Intervention
Source: PLoS Med. 2006 Nov 7;3(11):e433. doi: 10.1371/journal.pmed.0030433 (PMC1630714; doi:10.1371/journal.pmed.0030433)
Supplement: Supplementary file 1 [file pmed.0030433.sd001.doc]

Manejo de Internet por adolescentes ugandeses: Implicaciones para intervenciones a la VIH

**Resumen**

# Antecedentes. Se está reconociendo cada vez más al Internet como un método poderoso y sin costos mayores para brindar programas de intervenciones de salud a cantidades grandes de jóvenes por regiones geográficas diversas. No se sabe la viabilidad y accesibilidad de intervenciones a la salud por el Internet en lugares de escasos recursos, las cuales son las más necesitadas.

# Metodología y Resultados. “Media de Uganda y Tú” fue una encuesta sobre uso de Internet por adolescentes en Mbarara, Uganda, un municipio que sirve a una población rural en África del sub-Sahara. Se identificó al azar a participantes de estudiantes que asisten a una de cinco escuelas secundarias (internadas y externadas) en Mbarara, Uganda. Noventa y tres por ciento de los estudiantes identificados participaron (N=500).

Cuarenta y cinco por ciento (N=223) de los respondedores indicaron que han utilizado el Internet, 78% (N=175) de quienes indicaron que han utilizado el Internet en la semana pasada. La probabilidad de uso de Internet por adolescentes incrementa con educación materna. Casi dos de cinco respondedores (38%, N=189) indicaron que ya han utilizado una computadora o el Internet para buscar información sobre salud. Treinta y cinco por ciento (N=173) han utilizado la computadora o Internet para buscar información sobre VIH/SIDA y 20% (N=102) han buscado información sobre salud sexual. De usuarios del Internet, buscar información sobre VIH/SIDA por computadora o en línea fue relacionado a un nivel significativo al utilizar el Internet cada semana, enviar email, visitar a sitios de “chat,” y juegos en línea. Por otro lado, utilizar el Internet fue relacionado al revés al buscar información sobre VIH/SIDA por tecnología. Si el Internet fuera gratuito, 66% (N=330) buscaría información sobre prevención de VIH/SIDA en línea.

**Conclusiones.** Tanto el deseo como el uso verdadero del Internet para buscar información sobre salud sexual y VIH/SIDA son altos por estudiantes de escuelas secundarias en Mbarara. El Internet puede ser una estrategia prometedora para brindar intervenciones que reduzcan riesgos a VIH/SIDA sin costos mayores en lugares de escasos recursos que tengan acceso aumentando al Internet.
